# Magnetically controlled capsule endoscopy in one-time gastro-small intestinal joint examination: a two-centre experience

**DOI:** 10.1186/s12876-022-02302-0

**Published:** 2022-05-04

**Authors:** Ya-Wei Liu, Yuan-Chen Wang, Jia-Hui Zhu, Xi Jiang, Wei Zhou, Jie Zhang, Zhuan Liao, En-Qiang Linghu

**Affiliations:** 1 Department of Gastroenterology, The First Medical Center of Chinese PLA General Hospital/Chinese PLA Postgraduate Military Medical School, 28 Fuxing Road, Beijing, 100853 China; 2 National Clinical Research Center for Digestive Diseases, Department of Gastroenterology, Changhai Hospital, 168 Changhai Road, Shanghai, 200433 China

**Keywords:** Magnetically controlled capsule endoscopy, Gastric, Small intestine, Joint examination

## Abstract

**Background:**

The lesions of certain diseases are widely distributed in both stomach and small intestine, while the step-by-step strategy of gastroscopy followed by enteroscopy can be burdensome and costly. We aimed to determine if magnetically controlled capsule endoscopy (MCE) could be used in one-time gastro-small intestine (GSI) joint examination.

**Methods:**

In this study, data of patients in Chinese PLA General Hospital and Changhai Hospital who underwent MCE GSI examination from January 2020 to August 2021 were retrospectively analysed. The primary outcome of this study was the success rate of one-time GSI joint examination, and secondary outcomes included visualization and cleanliness of gastrointestinal tract, gastrointestinal transit times, diagnostic yield and safety of MCE examination.

**Results:**

A total of 768 patients were included. The success rate of one-time GSI joint examination was 92.58%. There were 94.92% MCEs observed > 90% gastric mucosa in the 6 anatomic landmarks. The rate of complete small bowel examination was 97.40%. The median gastric examination time, gastric transit time and small intestine transit time were 8.18 min, 63.89 min and 4.89 h, respectively. Magnetic steering of MCE significantly decreased gastric transit time (8.92 min vs. 79.68 min, *P* = 0.001) and increased duodenal lesion detection rate (13.47% vs. 6.26%, *P* = 0.001) when compared with non-magnetic steering group. Two capsules were retained and were removed by enteroscopy or spontaneously excreted.

**Conclusions:**

MCE is feasible to complete GSI joint examination and the detection of both gastric and small intestinal diseases can be achieved simultaneously.

*Trial registration* Clinical Trial Registration ClinicalTrials.gov, ID: NCT05069233.

**Supplementary Information:**

The online version contains supplementary material available at 10.1186/s12876-022-02302-0.

## Background

Since the first capsule endoscopy (CE) was introduced to the public in 2001, it has become the preferred examination for small intestine mucosal imaging because it is non-invasive, accurate and comfortable [1]. Recently, the diagnostic fields of CE have expanded to upper and lower gastrointestinal disorders due to the invention of oesophageal CE [2] and colon CE [3]. Furthermore, to realize the complete visualization of stomach, magnetically controlled capsule endoscopy (MCE) was developed [4]. Previous studies have demonstrated that the MCE has equally favourable diagnostic accuracy as conventional endoscopy and it is widely used in clinical practice [5, 6]. With more than 10 h battery life, MCE enables a further examination of the small bowel after gastric examination [6, 7].

The lesions of certain diseases, such as obscure gastrointestinal bleeding [8], dual antiplatelet-induced gastrointestinal bleeding [9], nonsteroidal anti-inflammatory drugs (NSAIDs)-related diseases and cirrhotic portal hypertension [10], are widely distributed in both the stomach and the small intestine. Unfortunately, some previously missed or underestimated lesions at the initial gastroscopic evaluation have higher requirements for small intestine examination, while the combination of gastroscopy and enteroscopy can be burdensome and costly [11, 12]. Early small bowel CE increases the possibility of detecting above lesions with a higher diagnostic yield than small bowel barium, CT enteroclysis, angiography and push enteroscopy [10, 13]. However, the lack of steerable control of the traditional small bowel CE and the limited battery life impede the complete visualization of the stomach and the small intestine at one time [14, 15].

The MCE with an active locomotion system fills these gaps and has the potential to achieve gastro-small intestine (GSI) joint examination. Compared with traditional small bowel CE which is usually performed after negative finding of gastroscopy and colonoscopy, the MCE can examine the stomach and the small bowel at one time, simplifying the clinical examination process. Nowadays, the MCE has been well-established for investigation of both the stomach and the small intestinal diseases, such as upper abdominal pain, gastric cancer, and iron deficiency anaemia or melaena [16, 17]. So far, the MCE system has been approved by National Medical Products Administration (NMPA) of China as an established investigation modality for gastric and small bowel examinations in adults. Considering the efficacy of MCE in one-time GSI joint examination has never been discussed, we aimed to explore its success rate, diagnostic yield and visualization in this study.

## Method

### Patients and study design

This study was a two-centre, retrospective, descriptive research at Chinese PLA General Hospital and Changhai Hospital, which was approved by the Medical Ethics Committees of the two centres and registered at www.clinicaltrials.gov (NCT05069233). This study was conducted according to the Declaration of Helsinki and followed STROBE guideline [18].

In this retrospective study, data of patients in Chinese PLA General Hospital and Changhai Hospital who underwent MCE GSI examination from January 2020 to August 2021 were retrospectively analysed. The included GSI joint examination patients accounted for 30% of all MCE examinations in these two centres. Patients with incomplete basic information and imaging data, less than 18 years old or inadequate gastrointestinal preparation were excluded.

### MCE examination procedure

The MCE system (Ankon Technologies Co. Ltd., Shanghai, China) consisted of a swallowable CE  (11.8 × 27 mm), a guidance magnetic robot, a data recorder, and a computer workstation with ESNavi software. The capsule can work for more than 10 h, meeting the one-time examination needs of both the stomach and the whole small intestine. The images were captured and recorded at 0.5–6 frames per second (fps) with 480 × 480 ppi resolution [19].

Considering the GSI examination requires bowel preparation, patients were asked to ingest 2 L polyethylene glycol (PEG) (Wanhe Pharmaceutical Co. Ltd., China) on the night before the examination. As for gastric preparation, simethicone and repetitive position change were provided to improve gastric cleanliness [20, 21], and 800–1000 ml water was ingested to fill the stomach [16]. Patients with a standardized gastrointestinal preparation regimen for MCE were instructed to swallow the capsule in the left lateral decubitus position with a small amount of water. When the capsule entered the stomach, it was rotated and advanced to the fundus and cardiac regions, followed by the gastric body, angulus, antrum, and pylorus under magnetic control. This procedure was repeated twice in each patient to better visualise the gastric mucosa. After completing the gastric examination, if the pylorus was open, the capsule would be dragged to the duodenum and examined the duodenum under magnetic control. If the pylorus was not open or capsule did not pass through the pylorus under magnetic control, the capsule would directly be switched to “small intestine mode” with an adaptive capture rate of 0.5–6 fps, and patients were allowed to leave the hospital with the data recorder for further image collection [7]. After the capsule entering the small bowel, patients were allowed to drink clear liquids, and could eat small amount of solid food 2 h later.

### Retrospective review

All patients’ baseline information, including age, sex, body mass index (BMI), indications for MCE, and medical history was retrospectively collected from the MCE databases in these two centres. All examinations were performed by two endoscopists (W.Z. at Changhai Hospital, and J.Z. at Chinese PLA General Hospital) with experience of more than 500 cases of MCE operation. Patients were followed up for 2 weeks to confirm the capsule excretion. Each MCE video was independently and blindly interpreted by two other experienced gastroenterologists. In the case of a discrepancy in the interpretation of capsule findings between the two MCE readers, a central committee composed of two MCE experts would be resorted to for a final decision.

### Study outcomes and definition

#### Primary outcome

The primary outcome of this study was the success rate of the GSI joint examination under MCE, which was defined as the proportion of patients who had a complete visualization of both the stomach and the small bowel at one time. Complete visualization of the stomach was defined as the observation of > 90% of the mucosa in all of the six primary gastric anatomical landmarks (cardia, fundus, body, angulus, antrum, and pylorus) [21]. If the capsule reached the cecum within its battery life, it was defined as a complete examination of small intestine [22].

#### Secondary outcomes

The secondary outcomes included visualization of the gastrointestinal tract, cleanliness of the gastrointestinal tract mucosa, gastrointestinal transit times, diagnostic yield and safety outcome.

The visualization of the gastrointestinal tract was made up of oesophageal visualization, gastric visualization and small intestinal visualization. For the oesophageal visualization, we focused on the number of images captured for oesophageal mucosa and the success rate of Z-line viewing from one to four quadrants [23]. As for the gastric visualization, we focused on the complete overall visualization of the gastric mucosa in the 6 anatomic landmarks (cardia, fundus, body, angulus, antrum, and pylorus), and a 3-point grading scale was used: 1, poor (< 70% of the mucosa was observed), 2, fair (70%–90% of the mucosa was observed), and 3, good (> 90% of the mucosa was observed) [6]. Visualization of the small bowel was determined by the rate of complete small bowel examination and the percentage of time during which the small-bowel view was clear, defined as not obscured more than 50% of the screen view. The clear-viewing percentage of the total small-bowel transit time was assessed by a 4-point scale: less than 25%, 25–49%, 50–75%, and greater than 75% [24].

The cleanliness of the gastrointestinal tract was mainly affected by mucus and bubbles. Oesophageal cleanliness was classified by the amount of bubbles/saliva on the Z-line area and recorded as a 3-grade scale (0, no interference; 1, minor interference; 2, major interference) [25]. The gastric cleanliness was recorded by a 4-grade scale of 1–4, which means poor (1, large amount of mucus or foam residue), fair (2, considerable amount of mucus or foam present precluding a completely reliable examination), good (3, small amount of mucus and foam, but not enough to interfere with the examination), and excellent (4, no more than small bits of adherent mucus and foam) [26]. The cleanliness score of the small intestine was the same as that of the oesophagus, which ranged from 0 to 2 [27]. The small intestine was divided into the proximal, middle and distal small intestine according to the small intestine transit time (SITT).

For the gastrointestinal transit time, we defined oesophageal transit time (ETT), gastric transit time (GTT) and SITT as the time between the first oesophageal image and the first gastric image, the first gastric image and the first small intestine image, and the first small intestine image and the first large intestine image, respectively. The total recording time (TRT) was the time of the last picture taken by the capsule. Gastric examination time (GET) was defined as the time from the first gastric image to the end of the gastric examination under magnetic control. Pyloric transit time (PTT) was defined as the time from completion of the gastric examination under magnetic control to the MCE entering the duodenum [7]. Further, subgroup analyses for PTT and GTT in the duodenum were conducted according to whether MCE passed pylorus by magnetic guidance or spontaneously.

Detection of lesions included positive findings in oesophagus, stomach and small bowel. Safety was assessed by the occurrence of procedure-related adverse events during the 2-week follow-up period, including abdominal pain, bleeding, and capsule retention.

### Statistical analysis

According to the 89% success rate of the GSI joint examination rate in the pilot study, alpha level of 0.05 (two-tailed) and a 5% allowable total error rate, we calculated the sample size as 642 [28]. The quantitative data was described by the mean and standard deviation (SD) or the median and interquartile range (IQR). For the quantitative data that lack of a normal distribution, the Mann–Whitney test was used for comparisons. Categorical data were presented as frequencies (percentages). Differences in categorical variables between groups were compared using Chi square test. A two-sided *P* value of < 0.05 was considered to be statistically significant. Statistical analyses were performed with SPSS software (version 23.0, SPSS Inc., Chicago, Illinois).

## Results

### Patient characteristics

From January 2020 to August 2021, 768 patients (mean age 48.35 years, range 18–94 years; 53.78% male) were analysed in the study. The major indications for GSI joint examination were abdominal pain or distension (295/768, 38.41%), followed by physical examination (162/768, 21.09%), acid reflux (92/768, 11.98%), diarrhoea (55/768, 7.16%), melaena (42/768, 5.47%), constipation (31/768, 4.04%), elevated tumour markers for gastrointestinal malignancies (21/768, 2.73%), nausea or vomiting (19/768, 2.47%), and weight loss (18/768, 2.34%). The baseline characteristics and indications for GSI joint examination are summarized in Table 1.

**Table 1:** Patients’ characteristics and indications for GSI joint examination

Characteristics	Results, n (%)
*Baseline characteristics*	
Male	413 (53.78)
Age, y	48.35 ± 17.59
BMI, kg/m^2^	23.28 ± 3.64
*Indication for GSI*	
Abdominal pain or distension	295 (38.41)
Physical examination	162 (21.09)
Acid reflux	92 (11.98)
Diarrhoea	55 (7.16)
Melaena	42 (5.47)
Constipation	31 (4.04)
Elevated tumour markers for gastrointestinal malignancies	21 (2.73)
Nausea or vomiting	19 (2.47)
Weight loss	18 (2.34)
Others	33 (4.30)
History	
*Diabetes*	25 (3.26)
*Helicobacter pylori* infection	245 (31.90)
Dual antiplatelet therapy history	49 (6.38)
Abdominal surgery	62 (8.07)
Smoking history	151 (19.66)
Drinking history	148 (19.27)

GSI, gastro-small intestine; BMI, body mass index

### Success rate of GSI joint examination and safety

There were 729 (94.92%) MCEs successfully observed > 90% gastric mucosa in all the 6 anatomic landmarks, as 39 patients failed to complete stomach examination. Among these patients, 27 cases were due to poor gastric preparation and 12 cases were due to MCE passing through the pylorus within 4 min. The success rate of small intestinal examination was 97.40%, with MCE failing to reach the caecum in 20 patients. In addition, two patients failed to complete the stomach examination and small bowel examination at the same time. Overall, GSI joint examination was achieved in 711 patients, and the success rate was 92.58% (Table 2). Capsule retention in the small intestine occurred in 2 cases (0.26%); one was successfully retrieved by double-balloon enteroscopy, and the other was excreted spontaneously.

**Table 2:** Success rate of GSI joint examination

Examination	Success rate, n (%)
Gastric examination	729 (94.92)
Small intestinal examination	748 (97.40)
GSI joint examination	711 (92.58)

GSI, gastro-small intestine

### Visualization of the oesophagus, stomach and small intestine

Table 3 shows the results concerning the visualization of oesophagus, stomach and small intestine. Visualization of the oesophagus mucosa was achieved in 763 patients (99.35%). The Z-line was detected in 372 patients (48.44%). In the stomach, visualizations of the main anatomical landmarks were considered to be good in the cardia, fundus, body, angulus, antrum, and pylorus in 98.05%, 96.09%, 98.57%, 99.22%, 99.74%, and 99.74% of patients, respectively. For the small intestine, 77.21%, 20.83%, 1.82%, and 0.13% of patients were visualized more than 75%, 50–75%, 25–50%, and less than 25% clean small intestine mucosa, respectively.

**Table 3:** Visualization of oesophagus, stomach and small intestine

Location	n (%)
*Oesophagus*	
Visualization of oesophagus mucosa	763 (99.35)
Z-line visualization	372 (48.44)
4 quadrants	62 (8.07)
≥ 3 quadrants	87 (11.33)
2–3 quadrants	114 (14.84)
< 2 quadrants	109 (14.19)
*Stomach*	
Visualization of primary anatomical landmarks	
Cardia	753 (98.05)
Fundus	738 (96.09)
Body	757 (98.57)
Angulus	762 (99.22)
Antrum	766 (99.74)
Pylorus	766 (99.74)
*Small intestine*	
Visualization of small intestine mucosa	
< 25%	1 (0.13)
25–50%	14 (1.82)
50–75%	160 (20.83)
≥ 75%	593 (77.21)

The cleanliness of the oesophagus mucosa was considered as no interference in 443 patients (57.68%). Gastric cleanliness was recorded as 3.81 ± 0.48, 3.74 ± 0.54, 3.68 ± 0.57, 3.85 ± 0.44, 3.86 ± 0.39 and 3.94 ± 0.25 in the cardia, fundus, body, angulus, antrum and pylorus, respectively. Cleanliness score in the proximal, middle, and distal small intestine was 0.17 ± 0.39, 0.33 ± 0.53 and 0.72 ± 0.69, respectively (Additional file 1: Table S1).

### Transit time and examination time of MCE

The median ETT and GET of MCE were 17.00 s (IQR, 10.00–32.00 s) and 8.18 min (IQR, 6.59–10.35 min), respectively. The median PTT was 54.02 min (IQR, 20.13–93.01 min), and the median GTT was 63.89 min (IQR, 31.50–101.77 min). In the small intestine, the SITT of MCE was 4.89 h (IQR, 3.90–6.19 h). For the total examination, the median TRT was 12.84 h (IQR, 12.22–13.35 h) (Additional file 1: Table S2).

Transpyloric passage of MCE under magnetic control was successfully steered preformed in 193 patients (25.13%). PTT and GTT in the magnetic steering group (0.10 min [IQR, 0.02–2.44] and 8.92 min [IQR, 5.68–14.46]) were significantly shorter than those in the non-magnetic steering group (68.92 min [IQR, 40.50–102.45] and 79.68 min [IQR, 51.01–111.51]) (*P* < 0.0001 in both) (Additional file 1: Table S3).

### Diagnostic yields of MCE

**Fig. 1:**
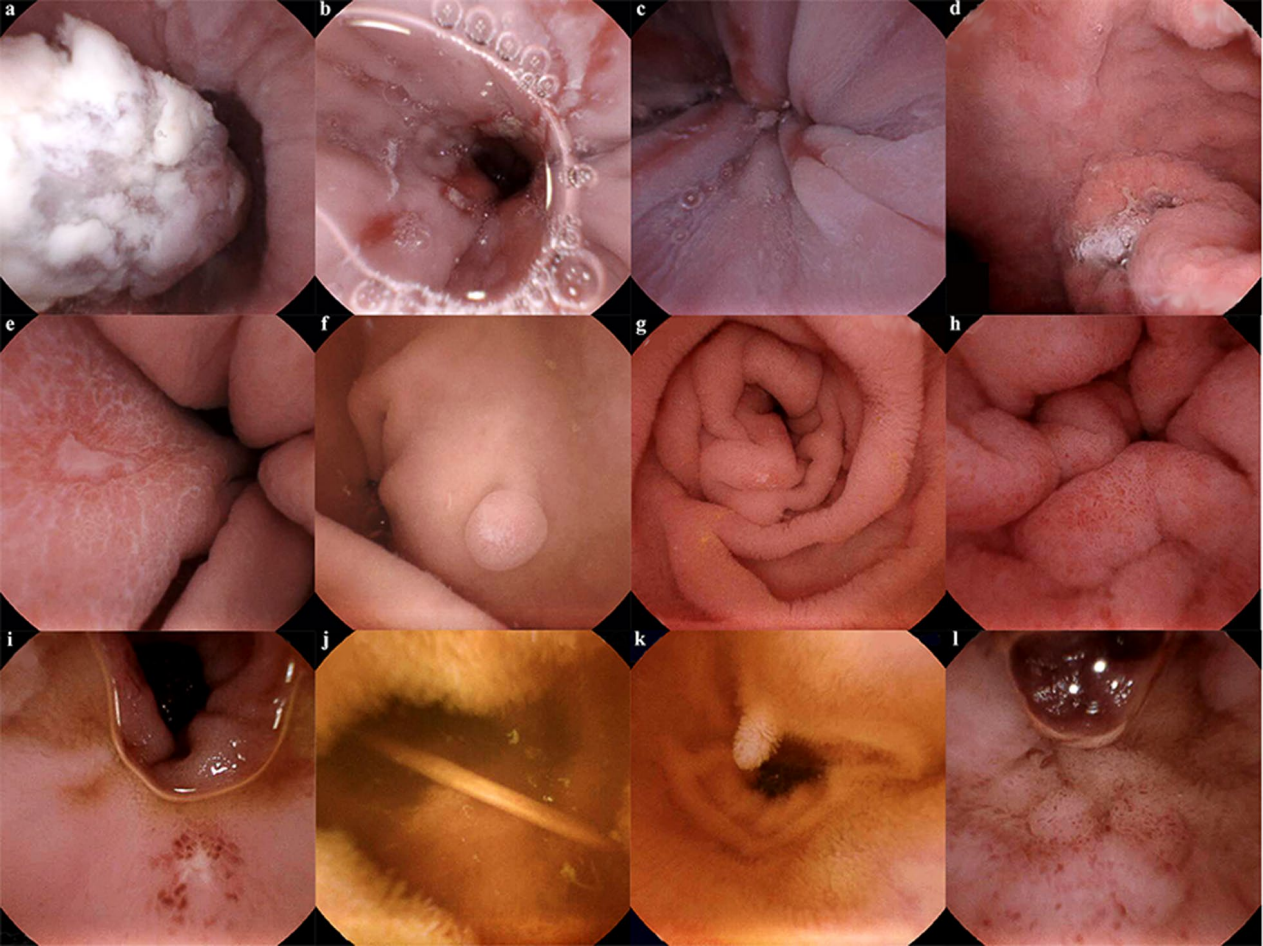
Positive lesions detected by MCE. **a** Oesophagus cancer. **b** Oesophagus ulcer. **c** Oesophagitis. **d** Gastric cancer. **e** Gastric ulcer. **f** Gastric polyp. **g** Duodenum ulcer. **h** Duodenitis. **i** Small intestine ulcer. **j** Parasite. **k** Small intestine polyp. **l** Small enteritis

The pathological lesions found by MCE in the GSI joint examination are shown in Table 4 and Fig. 1. Forty lesions (5.21%) were detected in the oesophagus, and oesophagitis was predominated (21/768, 2.73%). Lesions in the stomach, which predominated by gastritis (587/768, 76.43%) and gastric polyps (98/768, 12.76%), were found in 734 patients. Sixty-two patients (8.07%) with duodenal diseases were detected by MCE. The lesion detection rate of duodenal diseases in the magnetic steering group was higher than that in the control group (*P* = 0.001) (Additional file 1: Table S3). There were 134 patients diagnosed with small intestine diseases: including small intestinal ulcers (7.42%), enteritis (4.69%), vascular abnormalities (1.30%), submucosal lesions (1.17%), polyps (0.78%), inflammatory bowel disease (0.78%), parasites (0.52%) and diverticulum (0.39%).

**Table 4:** Positive findings in oesophagus, stomach, and small intestine

Lesion	Detected by MCE, n (%)
*Oesophagus*	40 (5.21)
Oesophagitis	21 (2.73)
Submucosal lesions	14 (1.82)
Oesophageal ulcer	2 (0.26)
Others*	3 (0.39)
*Stomach*	734 (95.57)
Gastritis	587 (76.43)
Gastric polyps	98 (12.76)
Submucosal lesions	25 (3.26)
Gastric ulcer	17 (2.21)
Tumour	1 (0.13)
Others^#^	6 (0.78)
*Duodenum*	62 (8.07)
Duodenitis	38 (4.95)
Duodenal ulcer	20 (2.60)
Others^&^	4 (0.52)
*Small intestine*	134 (17.44)
Small intestinal ulcer	57 (7.42)
Small enteritis	36 (4.69)
Vascular abnormalities	10 (1.30)
Submucosal lesions	9 (1.17)
Small intestinal polyps	6 (0.78)
Inflammatory bowel disease	6 (0.78)
Parasite	4 (0.52)
Diverticulum	3 (0.39)
Others^$^	3 (0.39)
*Positive findings in both the stomach and small intestine*	89 (11.58)

*Oesophageal venous tumour, oesophageal varices, oesophageal tumour

^#^Two cases of ectopic pancreas, 2 cases after gastric endoscopic submucosal dissection and 2 cases of vascular abnormalities in stomach

^&^Duodenal polyps in 2 cases, duodenal papilloedema in 1 case and duodenal submucosal eminence in 1 case

^$^One case of coeliac disease and 2 cases of small intestinal venous tumour

Eighty-nine cases (11.58%) of positive lesions were detected in both the stomach and the small intestine. Erosive gastritis combined with small enteritis occurred in 67 cases, and 4 cases involved inflammation in the upper gastrointestinal (UGI) tract and small intestine. Five patients suffered from both gastric polyps and small intestinal submucosal lesions. Ulcers in both the stomach and the small intestine were detected in 3 cases. Gastritis with small intestine diverticulum, gastritis with parasite, and gastritis with small intestinal vascular abnormality were all found in 3 patients.

## Discussion

This is the first study demonstrating that the MCE could be a feasible method for non-invasive one-time GSI joint examination, which provides new insights for gastrointestinal tract examination. Given that the combination of lesions in both the stomach and the small intestine is commonly seen, while the utilization of gastroscopy plus small bowel CE or enteroscopy is uncomfortable to patients and burdensome to medical resources, a one-time comfortable gastrointestinal tract examination procedure was the focus of this study. The invention of MCE fills these gaps and has been widely used in examination of gastrointestinal diseases, especially for gastric examination [15]. Previous studies reported the capability of MCE for gastric visualization; however, none of these studies illustrated the feasibility of MCE in joint examination from the stomach to the small intestine [29–32]. In this two-centre, retrospective study, MCE was successfully performed in all 768 patients and one-time GSI joint examination succeeded in 92.58% of patients, and no severe adverse event was observed.

For the oesophageal examination, the Z-line observation rate was only 48.43%, and 57.68% of patients with cleanliness of the oesophagus scored as grade 0. These results are insufficiently adequate to replace gastroscopy, which are similar to previous conventional capsule endoscopies [19, 33]. At present, indications for MCE examination do not include oesophageal disease and endoscopists could only describe the presentation of limited oesophageal pictures taken by the MCE in the final report. Previous studies provided the attachment of string to overcome the gravity and showed an increase in oesophageal examination time, improving diagnostic accuracy of oesophageal diseases [34–38]. The new method of detachable string MCE offers the possibility of complete viewing UGI and small intestine at one time.

For gastric examination, the present study showed that the incomplete visualization of gastric body, angulus, antrum and pylorus were only 1.43%, 0.78%, 0.26% and 0.26%, respectively. The satisfactory visualization rate of gastric mucosa owes to that the MCE was performed twice in a standardized operation and with a high level operator proficiency [19]. By using simethicone and repetitive position change, the gastric cleanliness in this study was considered good to excellent, which was similar to previous studies [20, 21]. Compared to the results of Ching HL et al. [30], our study showed that only 1.95% and 3.91% of patients had insufficient cardia and fundus examinations, respectively. The relevant poor visibility of MCE in the upper stomach was probably due to the mucus and bubbles being retained by the deep mucosal folds of the greater curvature [6, 20, 21]. Further, the “from fundus to antrum” examination steps in this study avoided incomplete visualization of the stomach [16], only 12 cases with rapid passage of MCEs.

In terms of the small intestine, the limited battery life often precludes complete examination. A previous meta-analysis illustrated that the small bowel incomplete examination rate was 12.08% [39], which is higher than our results. Various interventions, such as prolonging battery life, administering prokinetic agents, or endoscopically placement, were shown to contribute to the enhanced rate of completing small intestine examination [40–42]. More recent studies supported that the improvement of GTT by magnetic steering could improve the completion rate more efficiently [7, 12]. In this study, magnetic steered MCE in 193 cases significantly shortened the PTT and GTT, and enhanced the detection rate of lesions in the duodenum from 6.26 to 13.47%. With bowel preparation, our cleanliness of the small intestine was considered as being minor or having no interference with MCE visualization. Capsule retention is the most focused MCE adverse event and is usually treated by surgery, while in recent years the trends of endoscopic methods and medical treatments have increased [39]. Although two cases suffered from retention, none of them experienced gastrointestinal obstruction and excreted without surgery, suggesting that MCE has low adverse event rates and is safe for clinical application.

Another breakthrough of this study was the extended diagnostic field by one-time GSI joint examination. There were 89 cases with both stomach and small intestine diseases. Additionally, one case of oesophageal cancer and one case of gastric cancer were detected by MCE. These results highlighted the necessity of GSI joint examination to comprehensively evaluate gastrointestinal diseases, which can potentially be fulfilled by the MCE with satisfactory diagnostic ability.

There are limitations to this study. Firstly, this is a retrospective observational study suggesting that MCE is feasible for one-time GSI joint examination. Randomized controlled trials of MCE compared to gastroscopy plus enteroscopy or small bowel CE are warranted. Secondly, oesophageal examination is not satisfactory due to the limited ETT, further studies are needed to determine the feasibility of detachable string MCE in one-time UGI and small intestine joint examination. Further, we did not explore the GSI success rate and lesions detection rate in certain diseases that distribute throughout the whole gastrointestinal tract, such as dual antiplatelet-induced gastrointestinal bleeding and cirrhotic portal hypertension. Moreover, unlike traditional CE, MCE needs both operation and image interpretation. Thus, operator proficiency training is vital, and the high cost of MCE examination may limit the popularization of the MCE. Lastly, MCE is still a diagnostic technique, and its biopsy or therapeutic functions are under exploration, traditional endoscopies are necessary for patients who need further interventions.

This study demonstrated the feasibility and safety of MCE in one-time GSI joint examination. Early pyloric passage of MCE can also be benefited by magnetic steering, which enhanced the small intestine complete examination rate and increased the MCE diagnostic field. The favourable application of MCE to different parts of the gastrointestinal tract may have the potential to replace traditional endoscopy in certain scenarios. Further studies are required to achieve one-time overall gastrointestinal tract examinations.

## Supplementary Information

**Additional file 1: Table S1**. Cleanliness of oesophagus, stomach and small intestine. **Table S2**. Examination times of MCE in oesophagus, stomach, and small intestine. **Table S3**. Comparation of manipulation related parameters and detection of duodenal lesions.

## Data Availability

The datasets generated during the current study are not publicly available due to confidentiality of human subjects but are available from the corresponding author on reasonable request.

## Abbreviations

MCE: Magnetically controlled capsule endoscopy
GSI: Gastro-small intestine
CE: Capsule endoscopy
NSAID: Nonsteroidal anti-inflammatory drug
PEG: Polyethylene glycol
BMI: Body mass index
SITT: Small intestine transit time
ETT: Oesophageal transit time
GTT: Gastric transit time
TRT: Total recording time
GET: Gastric examination time
PTT: Pyloric transit time
SD: Standard deviation
IQR: Interquartile range
UGI: Upper gastrointestinal
