# Supplementary material for: Magnetically controlled capsule endoscopy in one-time gastro-small intestinal joint examination: a two-centre experience
Source: BMC Gastroenterol. 2022 May 4;22:222. doi: 10.1186/s12876-022-02302-0 (PMC9069740; doi:10.1186/s12876-022-02302-0)
Supplement: Supplementary file 1 — Additional file 1: Table S1. Cleanliness of oesophagus, stomach and small intestine. Table S2. Examination times of MCE in oesophagus, stomach, and small intestine. Table S3. Comparation of manipulation related parameters and detection of duodenal lesions. [file 12876_2022_2302_MOESM1_ESM.docx]

**Additional file**

**Additional file 1: Table S1. Cleanliness of oesophagus, stomach and small intestine**

| **Location** | **Cleanliness Score** |
| --- | --- |
| Oesophagus (distribution of cleanliness grade) |  |
| Grade 0, n (%) | 443 (57.68) |
| Grade 1, n (%) | 268 (34.90) |
| Grade 2, n (%) | 57 (7.42) |
| Stomach (mean grade in each anatomic landmark) |  |
| Cardia | 3.81±0.48 |
| Fundus | 3.74±0.54 |
| Body | 3.68±0.57 |
| Angulus | 3.85±0.44 |
| Antrum | 3.86±0.39 |
| Pylorus | 3.94±0.25 |
| Small intestine (mean grade in each segment of the small intestine) |  |
| Proximal small intestine | 0.17±0.39 |
| Middle small intestine | 0.33±0.53 |
| Distal small intestine | 0.72±0.69 |

Oesophageal and small intestine cleanliness was recorded as a 3-grade scale (0, no interference; 1, minor interference; 2, major interference).

The gastric cleanliness was recorded by a 4-grade scale of 1 to 4 (1, large amount of mucus or foam residue; 2, considerable amount of mucus or foam present precluding a completely reliable examination; 3, small amount of mucus and foam, but not enough to interfere with the examination; 4, no more than small bits of adherent mucus and foam).

**Additional file 1: Table S2. Examination times of MCE in oesophagus, stomach, and small intestine**

| **Variables** | **Examination time** |
| --- | --- |
| Median ETT, s (IQR) | 17.00 (10.00-32.00) |
| Median GET, min (IQR) | 8.18 (6.59-10.35) |
| Median PTT, min (IQR) | 54.02 (20.13-93.01) |
| Median GTT, min (IQR) | 63.89 (31.50-101.77) |
| Median SITT, h (IQR) | 4.89 (3.90-6.19) |
| Median TRT, h (IQR) | 12.84 (12.22-13.35) |

ETT, oesophageal transit time; GET; Gastric examination time; PTT, Pyloric transit time; GTT, gastric transit time; SITT, small intestine transit time; TRT, total recording time; IQR, interquartile range.

**Additional file 1: Table S3. Comparation of manipulation related parameters and detection of duodenal lesions**

| **Variables** | **Magnetic steering group (n=193)** | **Non-magnetic steering group (n=575)** | ***P*** |
| --- | --- | --- | --- |
| Median PTT, min (IQR) | 0.10 (0.02-2.44) | 68.92 (40.50-102.45) | 0.000 |
| Median GTT, min (IQR) | 8.92 (5.68-14.46) | 79.68 (51.01-111.51) | 0.000 |
| Detection rate of lesions in duodenum | 13.47% (26/193) | 6.26% (36/575) | 0.001 |
